# Spatiotemporal neural network with attention mechanism for El Niño forecasts

**DOI:** 10.1038/s41598-022-10839-z

**Published:** 2022-05-03

**Authors:** Jinah Kim, Minho Kwon, Sung-Dae Kim, Jong-Seong Kug, Joon-Gyu Ryu, Jaeil Kim

**Affiliations:** 1 Coastal Disaster Research Center, Korea Institute of Ocean Science and Technology, Pusan, 49111 South Korea; 2 Division of Environmental Science and Engineering, Pohang University of Science and Technology, Pohang, 37673 South Korea; 3 Satellite Wide area Infra Research Section, Electronics and Telecommunications Research Institute, Daejeon, 34129 South Korea; 4 School of Computer Science and Engineering, Kyungpook National University, Daegu, 41566 South Korea

**Keywords:** Projection and prediction, Physical oceanography

## Abstract

To learn spatiotemporal representations and anomaly predictions from geophysical data, we propose *STANet*, a spatiotemporal neural network with a trainable attention mechanism, and apply it to El Niño predictions for long-lead forecasts. The *STANet* makes two critical architectural improvements: it learns spatial features globally by expanding the network’s receptive field and encodes long-term sequential features with visual attention using a stateful long-short term memory network. The *STANet* conducts multitask learning of Nino3.4 index prediction and calendar month classification for predicted indices. In a comparison of the proposed *STANet* performance with the state-of-the-art model, the accuracy of the 12-month forecast lead correlation coefficient was improved by 5.8% and 13% for Nino3.4 index prediction and corresponding temporal classification, respectively. Furthermore, the spatially attentive regions for the strong El Niño events displayed spatial relationships consistent with the revealed precursor for El Niño occurrence, indicating that the proposed *STANet* provides good understanding of the spatiotemporal behavior of global sea surface temperature and oceanic heat content for El Niño evolution.

## Introduction

The El Niño Southern Oscillation (ENSO) is a cycle of warm and cold phases of sea surface temperature (SST) in the equatorial Pacific Ocean that influences extreme weather and ocean events with significant impacts, such as high temperatures, cold waves, heavy rain, and marine heatwaves^1–4^. Meteorological and marine disasters cause serious damage and loss to society as a whole, including the environment and economy, so accurate and prompt forecasts of climate signals such as Nino, are necessary.

In general, dynamic numerical models are used to forecast ENSO, and statistical models are also used to predict ENSO by analyzing historical data^5–8^. With the recent rise in the use of machine learning, particularly deep learning, various machine learning techniques^9,10^ and deep neural networks^11–15^ are used to predict El Niño, and they demonstrated good predictability, compared to conventional numerical and statistical methods.

Among deep learning-based approaches, a two-dimensional convolutional neural network, which trained using the Coupled Model Intercomparison Project Phase 5 (CMIP5) model simulation data^16^ (denoted as *2D CNN* hereafter), showed the best performance with a correlation skill of 0.5 or more for 14-month forecast lead with 3-month sequence’s two inputs of SST and oceanic heat content (HC) anomalies^12,15^. However, the *2D CNN* has limitations in learning the contextual changes of SST and HC anomalies over time as well as their spatial characteristics over a large area, because the *2D CNN* only learns the relevant features of SST and HC anomalies at individual time-points separately for the ENSO prediction.

To be concerned with the sequential characteristic of El Niño  occurrence, a recurrent neural network (RNN)^17^ with 2D convolutional layers has been introduced to learn temporal patterns from the high-level representations of SST anomalies^11^. They obtained a correlation coefficient of approximately 0.85 for the 6-month forecast lead using sequential input of continuous 24-month SST anomaly time series. Convolutional long short-term memory (LSTM) network^18^ has been employed to encode the long-term dependency of the temporal sequential features for El Niño prediction^13,14^. However, the correlation coefficients for the 12-month forecast lead were approximately 0.38 and 0.60, showing lower performance than the *2D CNN* using only local spatial features. Therefore, the *2D CNN* model^12,15^ showed superior performance to the dynamic model and various deep learning models with the best forecasting skills for the first 6 forecasting lead months. The correlation skill of the Nino3.4 index in the *2D CNN* model is above 0.5 for a lead of up to 17 months, whereas it is 0.37 at a lead of 17 months in the SINTEX-F5, a leading dynamic forecasting system. And the *2D CNN* provides a skillful forecast of ENSO events up to 1.5 years in advance. Therefore, the unified *2D CNN* model^15^, which is the state-of-the-art (SOTA) method, is implemented as the baseline model and is compared with the results of the model proposed in our study.

Spatiotemporal forecasting using geophysical data has its own specificity, such as high-dimensional, often limited in scope, and temporally correlated, which should be considered in building deep neural networks. In this study, we propose *STANet*, a spatiotemporal neural network with a trainable attention mechanism that can learn the characteristics of spatiotemporal geophysical data over time and test its predictability by applying it to El Niño forecasts. In particular, through the effective receptive field and dilated convolution^19^ as well as attention mechanism in recurrent neural network^20^, it is possible to obtain greater understanding of the spatiotemporal relationship between global-scale SST and HC anomalies that affect the occurrence of El Niño. In contrast to the *2D CNN*, which does not consider any temporal order of given input, the proposed *STANet* employs the 3D receptive field blocks with 2D + time convolution filters to learn spatiotemporal patterns from the short-term input (three months) and the stateful LSTM module with an attention mechanism to learn the temporal order of the long-term sequences. The receptive field block efficiently increases the receptive field using dilated convolution layers and residual skip-connection, and it allows the model to learn spatiotemporal features from larger regions. The stateful LSTM preserves long-term contextual information across sequential input data by keeping its hidden state and cell state even after mini-batch inference. In addition, the attention mechanism allows the proposed model to process the geophysical data and focus on more relevant regions at specific time steps precisely in the forecasting task. To examine the spatiotemporal feature representation capability according to the architectural characteristics of *STANet*, ablation experiments are performed through the combination of individual network modules of the proposed *STANet* for the El Niño prediction task. In addition, to evaluate how well the *STANet* understand the spatiotemporal behavior of SST and HC for El Niño prediction, we will look into whether the trained attention map and the precursor for El Niño occurrence are consistent.

## Results

### Overall predictability

**Figure 1:**
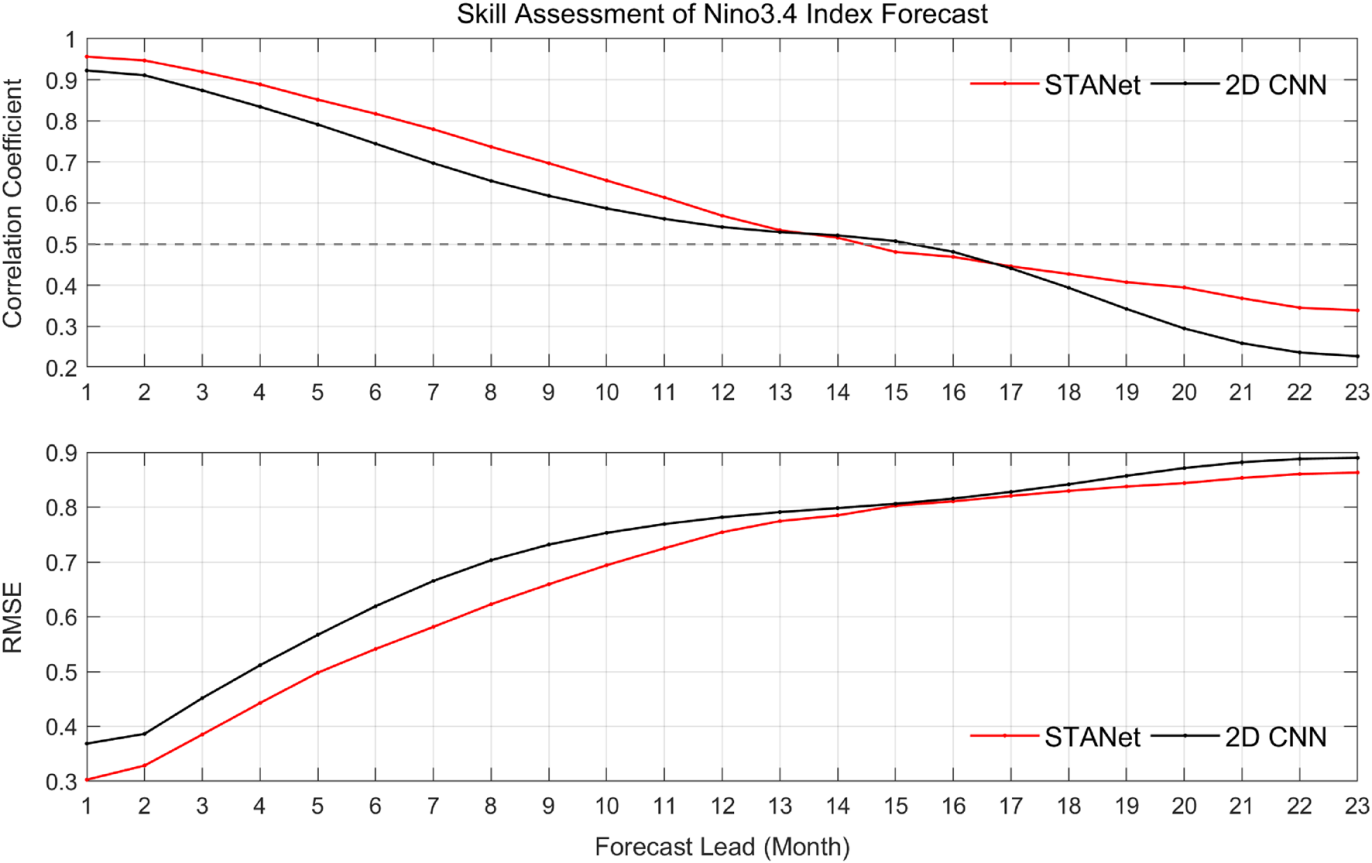
Time series of correlation coefficient (*CC*) and the root-mean-square-error (*RMSE*) for 23-month forecast lead time of the proposed *STANet* and the state-of-the-art *2D CNN* model for the test period of 1980–2017.

**Figure 2:**
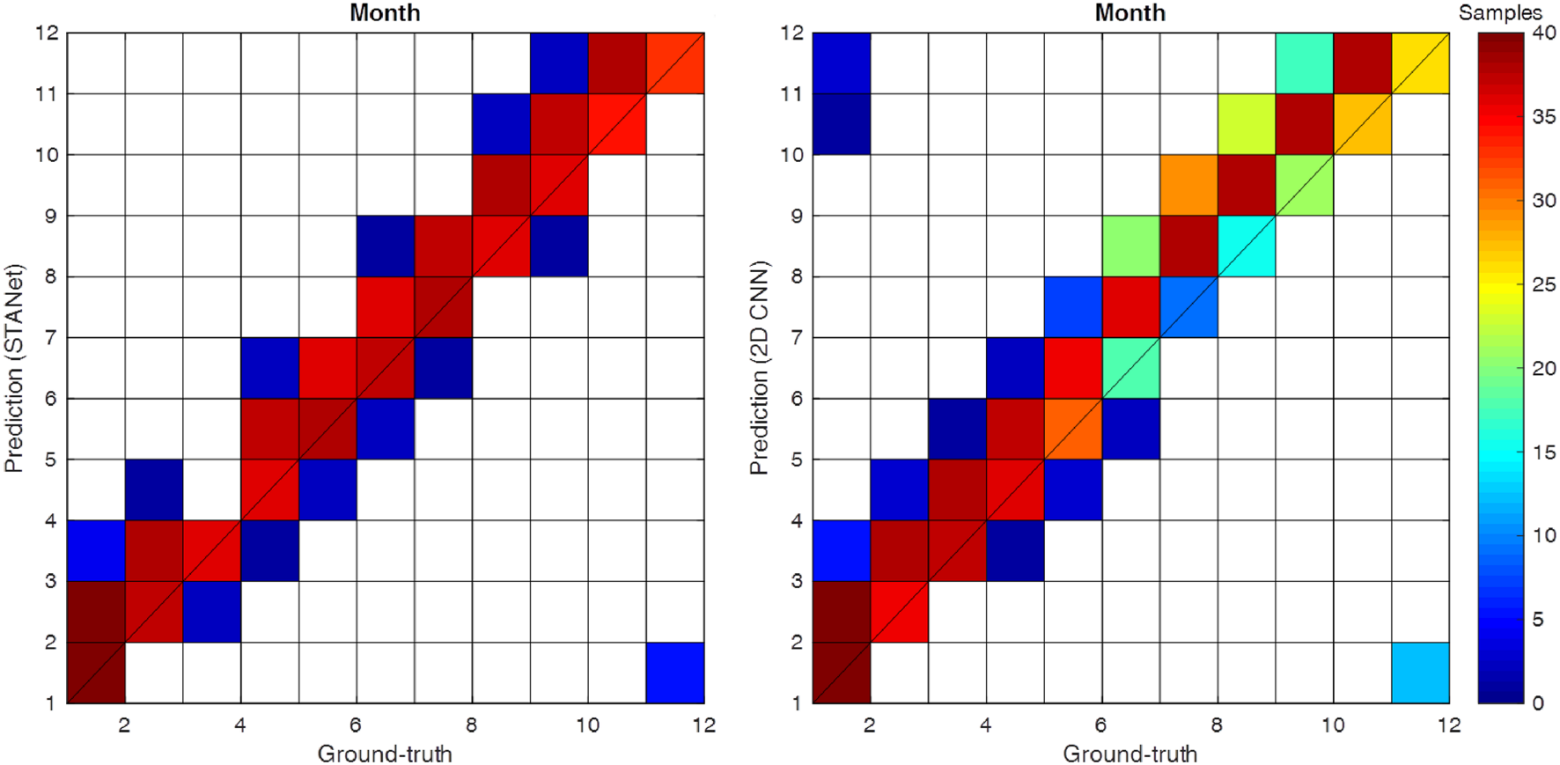
Density scatter of comparison between the ground-truth and the corresponding calendar month classification results from *STANet* (left panel) and the state-of-the-art *2D CNN* model (right panel).

Figures 1 and 2 show the time series of the correlation coefficient (*CC*), root-mean-square-error (*RMSE*), and density scatter of the calendar month classification to evaluate the performance of the proposed *STANet* and the SOTA model (*2D CNN*) in El Niño prediction for the 23-month forecast lead time from 1980 to 2017. The red and black lines represent the *STANet* and *2D CNN* model, respectively. The overall El Niño predictability of the proposed *STANet* was improved for the 12-month forecast lead time with the *CC* improving by 5.8% and the *RMSE* decreasing by 0.07 on average compared to the SOTA model. Classification accuracy was also improved by 13% during the entire test period (see Figs.  2, 7).

**Figure 3:**
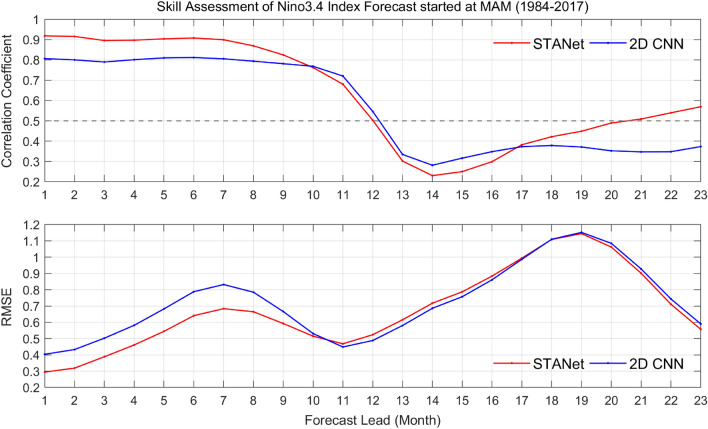
Time series of of correlation coefficient (*CC*) and the root-mean-square-error (*RMSE*) for 23-month forecast lead time of the proposed *STANet* and the state-of-the-art *2D CNN* model for forecasts employing input data from the March–April–May (MAM) season.

### Spring predictability barrier

The spring predictability barrier, a unique feature of ENSO^21^ means that ENSO forecasting accuracy decrease dramatically during boreal spring when an ENSO event begins to grow^8,22^. To investigate the predictability for forecasts initiated in boreal spring, the forecasts with input anomalies during the March–April–May (MAM) season were also performed using the proposed *STANet* and *2D CNN*. The comparison results are shown in Fig 3. The red and blue lines represent the time series of *CC* and *RMSE* for the proposed *STANet* and SOTA *2D CNN* model’s 23-month forecast lead, respectively. The *CC* of the Nino3.4 index predicted up to 9 months of the forecast lead time was improved up to 10% and the *RMSE* decreased by approximately 0.1 or more. The experimental results show that the proposed *STANet* was trained to learn the temporal features of global SST and HC behavior for the occurrence of El Niño, specially the long-term temporal dependency from sequential data.

### Time-series prediction

To test the spatiotemporal feature representation capability of the *STANet* based on its architectural characteristics, ablation experiments were carried out using the proposed *STANet*’s network modules for the El Niño prediction task. In addition, to examine how well the *STANet* understands the spatiotemporal behavior of SST and HC for El Niño prediction, we will look into the consistency of the trained attention map and precursor for El Niño occurrence.

**Figure 4:**
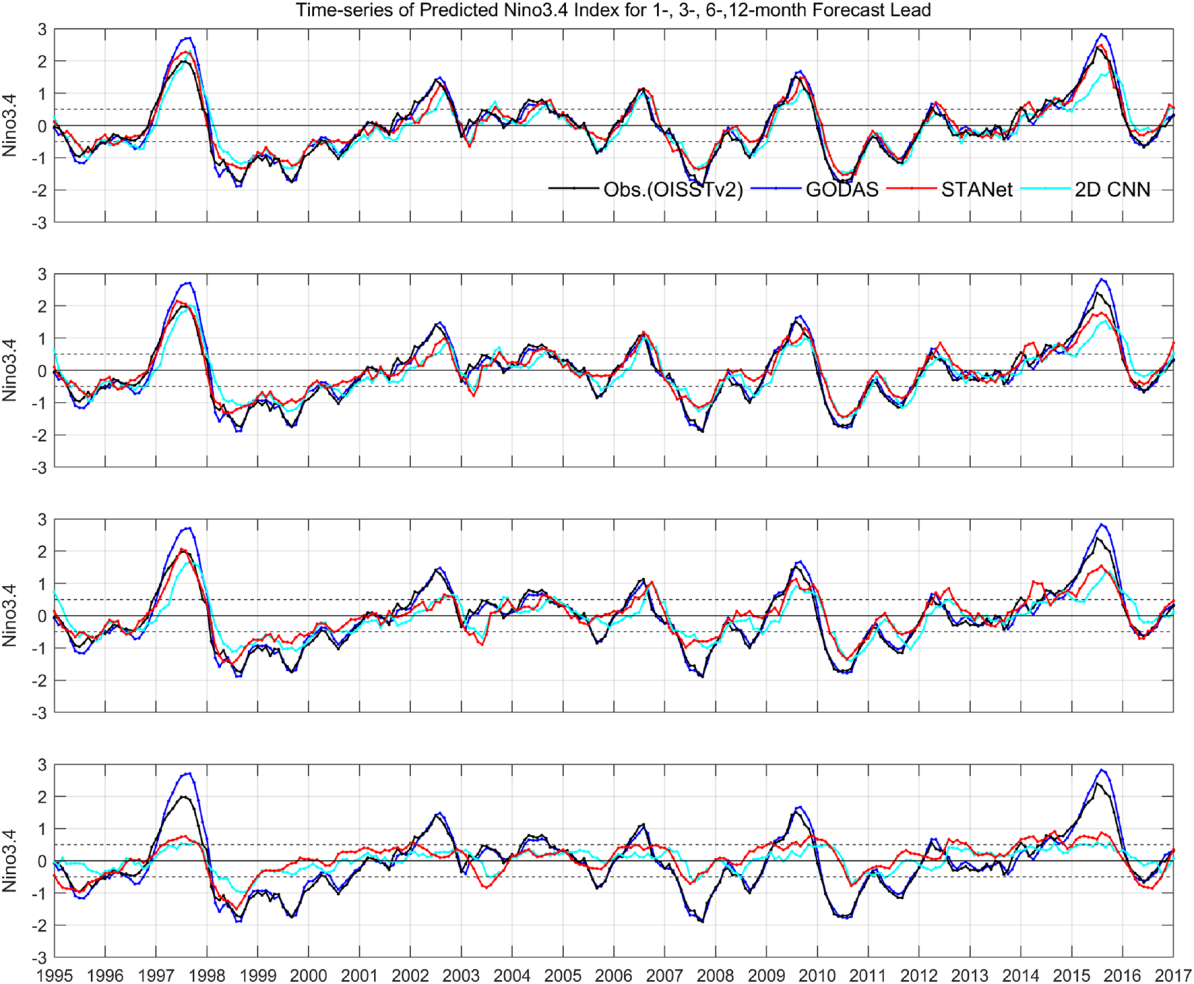
Time series of predicted Nino3.4 index from the proposed *STANet* with observation (OISST v2), GODAS, and SOTA *2D CNN* result for test period of 1996–2016. From top to bottom, the time series with forecast lead time of 1-, 3-, 6-, and 12-month are shown, respectively.

**Figure 5:**
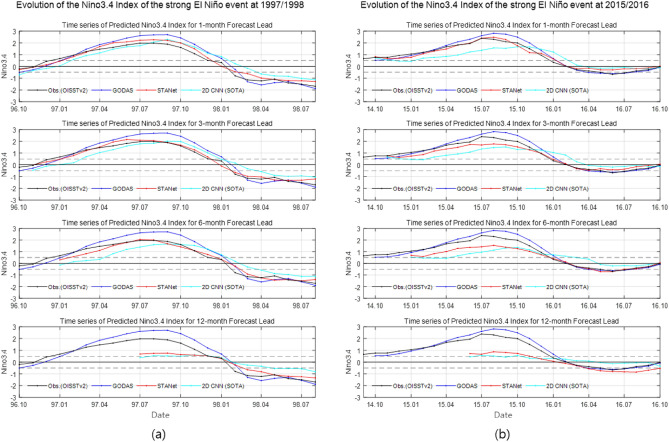
Evolution of the Nino3.4 index for two strong El Niño events of (**a**) 1997/1998 and (**b**) 2015/2016. From top to bottom, the time series with forecast lead time of 1-, 3-, 6-, and 12-month are shown, respectively.

The following Fig. 4 shows the Nino3.4 time series over the test period of 1996–2016 to examine how much the predictability of strong El Niño events was improved in terms of Nino3.4 indices. In addition, Fig. 5 shows the evolution of the Oceanic Nino Index during the two strong El Niño events of 1997/1998 and 2015/2016 through the time series of the Nino3.4 index, respectively. In Figs. 4 and 5, the red line represents the predicted time series of the proposed *STANet*, and the black, blue, and turquoise lines represent OISST v.2 observations, GODAS test data, and the predicted time series of the SOTA *2D CNN* model, respectively. From top to bottom, time series with forecast lead times of 1, 3, 6, and 12 months are shown. As the forecast lead time increases, the peak value is underestimated, and it is difficult to expect predictability concerning the 12 forecast lead month. When the peaks of the three main El Niño events for 1997/1998, 2009/2010, and 2015/2016 are plotted in time series for the 1- to 6-month forecast lead time, it is clear that the proposed *STANet* model matches the peak Nino3.4 index as well as the time phase better than the SOTA *2D CNN* model.

### Interpretation of the precursor of ENSO with attention maps

**Figure 6:**
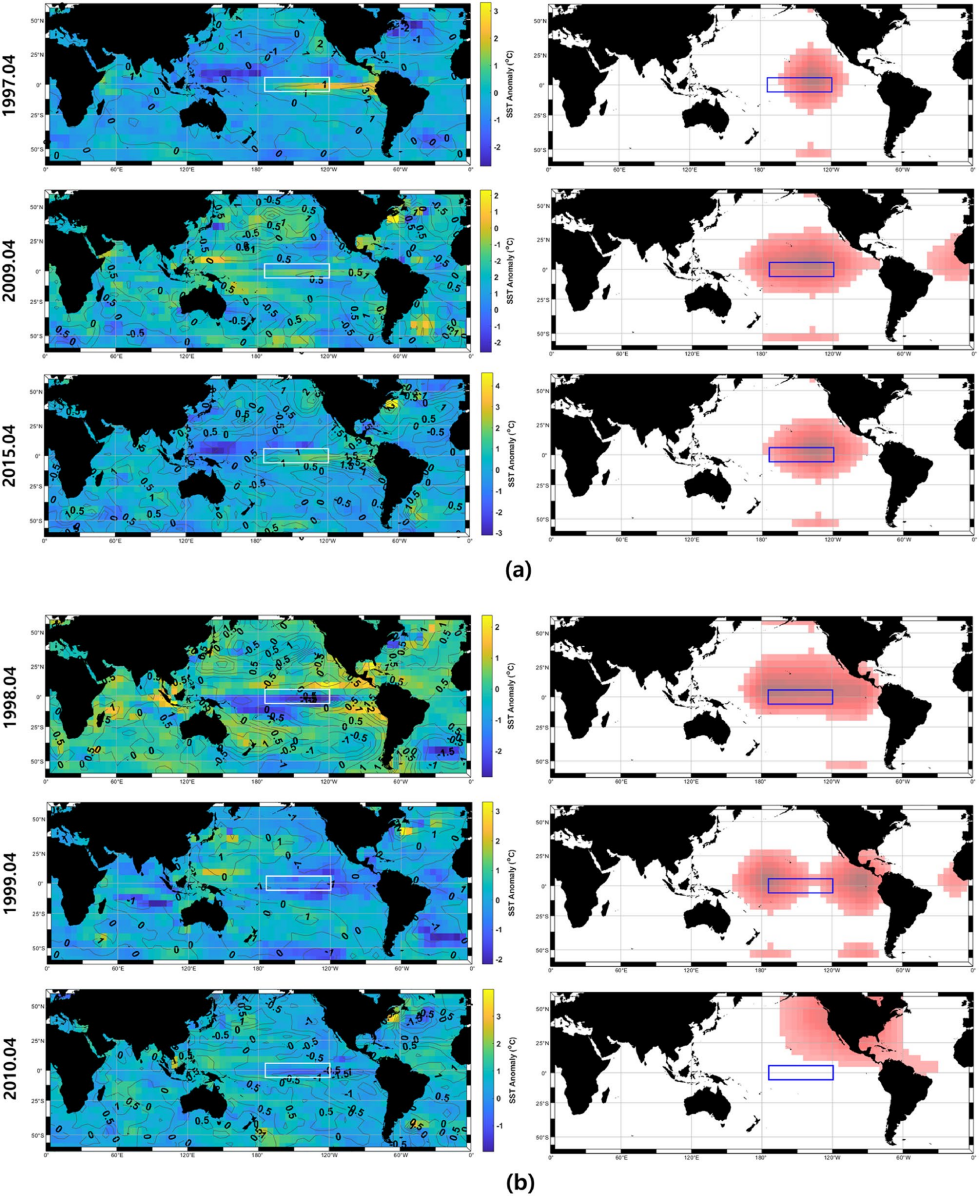
Visualization of trained attention maps that acted as precursors to representative (**a**) three El Niño events of 1997/1998, 2009/2010, and 2015/2016 (top to bottom) and (**b**) three La Niña events of 1998/1999, 1999/2000, and 2010/2011 (top to bottom). Shaded backgrounds and contours are spatial distributions for SST and HC anomalies several months prior to the event occurrence (see each figure title at the far left of the picture) used as inputs for El Niño and La Niña predictions in the left column of the figure. And the red gradient area in the right column of the figure shows the region where the proposed *STANet* is most affected by the input data given for Nino3.4 prediction. And the white and blue boxes show the region of Nino3.4. This figure is generated using Matlab R2018b (https://kr.mathworks.com/products/matlab.html).

Furthermore, Fig. 6 visualizes the spatially attentive regions, referred to as a trained attention map for strong three El Niño events of 1997/1998, 2009/2010, and 2015/2016 and three La Niña events of 1998/1999, 1999/2000, and 2010/2011^23^. In Fig. 6 of trained attention maps that acted as precursors to strong ENSO events, shaded backgrounds and contours are spatial distributions for SST and HC anomalies several months prior to the event occurrence (see each figure title) used as inputs for El Niño and La Niña predictions, and the legends of SST and HC anomalies are displayed on the right and overlaid contours of individual figures, respectively. And the white and blue squares show the region of Nino3.4, and the red gradient on the right panels shows the region where the proposed *STANet* is most affected by the input data given for Nino3.4 prediction.

A spatial region of attention is selected from among the whole area of spatial data, and values in this region are further processed with a higher weight than the rest of the spatial data. As a result, the attention map in which a specific area is highlighted in red indicates the area that had the most influence on the ENSO event. The attention maps clearly show where the SST and oceanic HC are important for a future ENSO evolution. The attention maps for the three El Niño years, for examples, consistently show that the central Pacific HC anomalies at the onset phase of the El Niño are a primary precursor for El Niño development, as suggested by the recharge oscillator theory^24,25^.

In addition, the northern hemisphere off-equatorial SST is an important precursor of El Niño development^26^, which seems to be captured in the attention map. Interestingly, the attention map for the 2009 El Niño is somewhat different from the other two events. That is, the attention map located to some extent in the central Pacific, and there is a clear signal in the northern tropical Atlantic Ocean, which may be related to the fact that the 2009 El Niño is a central Pacific-type El Niño^27^.

The attention map for the La Niña events also tends to capture the equatorial Pacific signals according to the recharge oscillator^24,25^. However, for the 2010 La Niña events, the eastern Pacific and Atlantic warm pools are attentive regions, which may be related to the connection with the Atlantic warm pool^28^.

The trained attention in this way shows spatial relationships consistent with the revealed El Niño dynamics, indicating that the proposed *STANet* provides a good understanding of the spatiotemporal behavior of the global SST and oceanic HC for El Niño prediction. In other words, the proposed *STANet* can determine which area of the input SST and oceanic HC anomalies to focus on for El Niño forecasting.

### Ablation experiments

**Figure 7:**
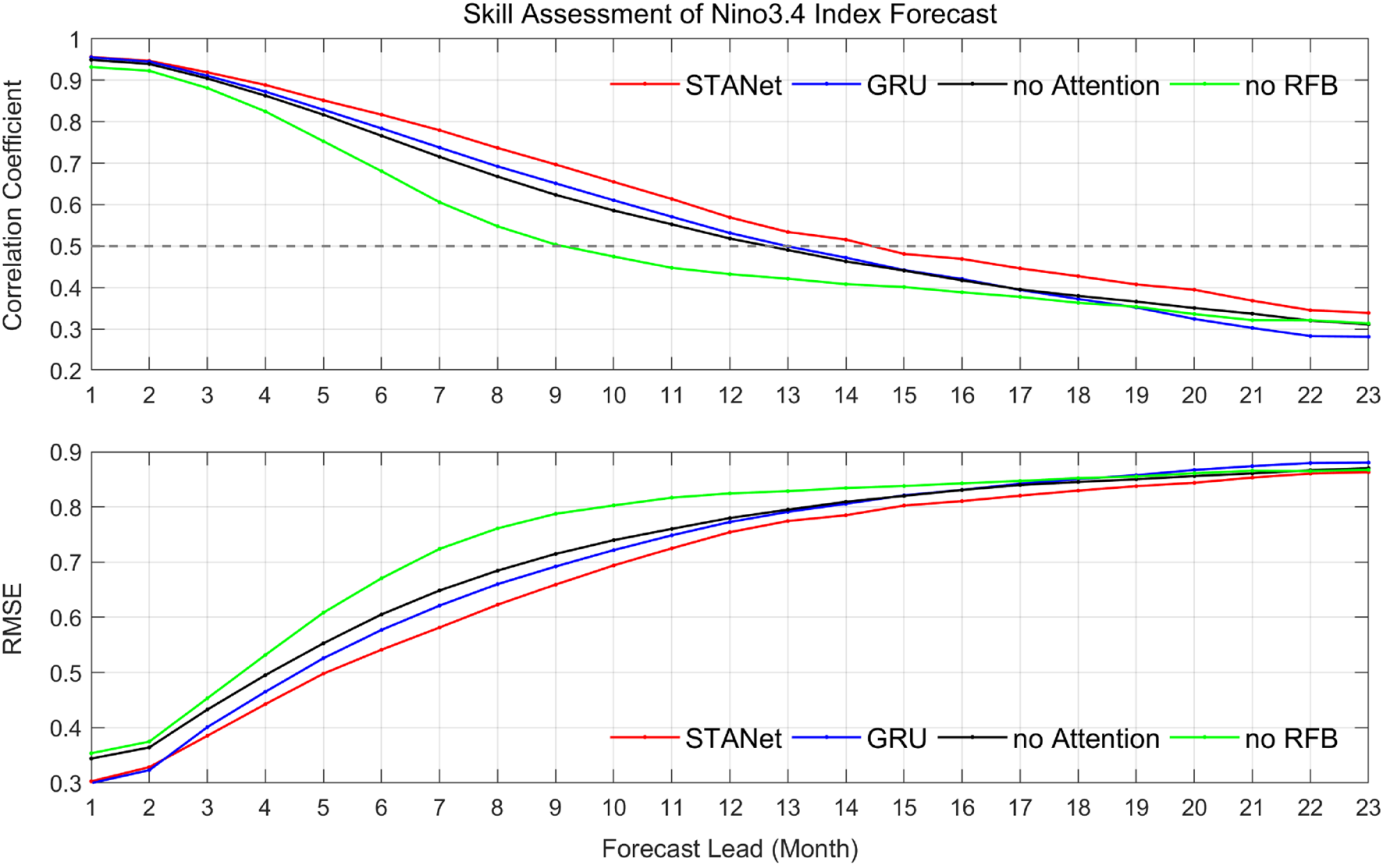
Time series of correlation coefficient (*CC*) and the root-mean-square-error (*RMSE*) for 23-month forecast lead time of the proposed *STANet* with 3 cases of ablation experiments for the test period of 1980–2017. “*STANet*” is the proposed architecture. “GRU” is an alternative model using GRU module^29^ instead of the LSTM module. “no Attention” indicates the *STANet* without attention mechanism. “no RFB” indicates the *STANet* using 3D convolution layers instead of the receptive field blocks. Lower RMSE and higher CC indicate better performance in Nino3.4 index prediction.

**Figure 8:**
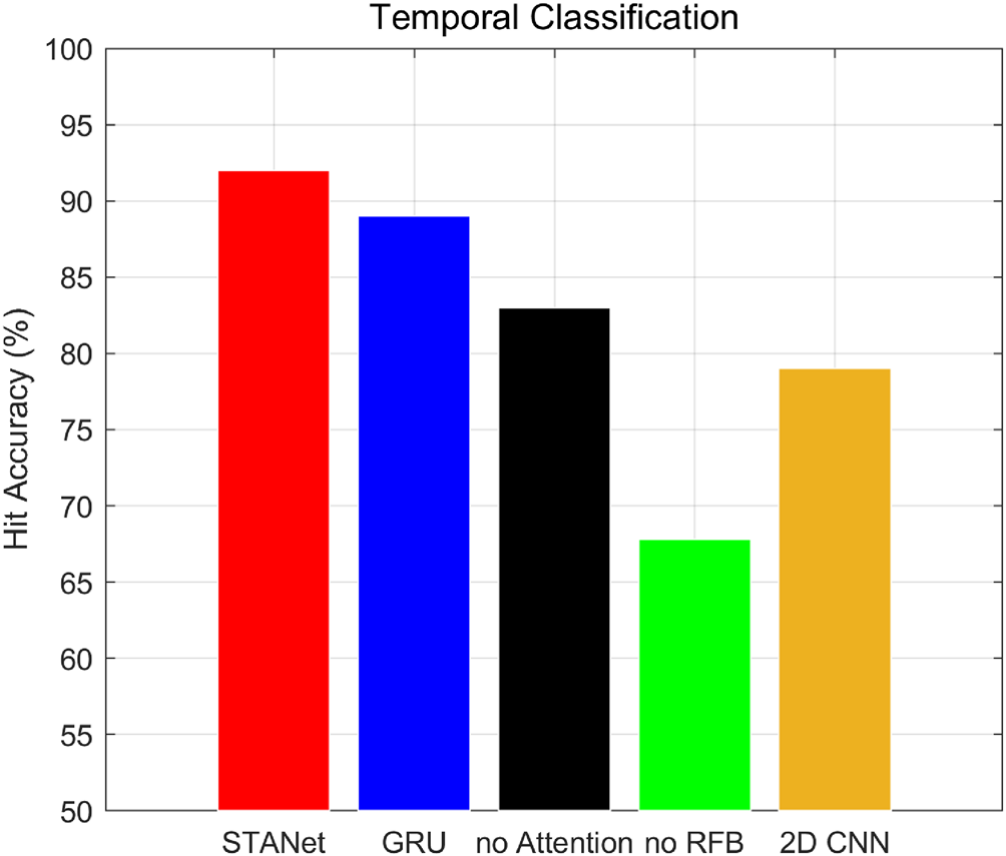
Hit accuracy for the calendar month classification for the proposed *STANet* with 3 cases of ablation experiments and the SOTA *2D CNN* for the test period of 1980–2017. “*STANet*” is the proposed architecture. “GRU” is an alternative model using GRU module^29^ instead of the LSTM module. “no Attention” indicates the *STANet* without attention mechanism. “no RFB” indicates the *STANet* using 3D convolution layers instead of the receptive field blocks.

Figures 7 and 8 show the comparative results of ENSO predictability based on the deep neural network’s architectural features (refer to the ablation experiments in Table 2). Each figure shows the time series of *CC* and *RMSE* on Nino3.4 index prediction for the 23-month forecast lead time and the hit accuracy of the calendar month classification for the test period of 1980–2017. In comparison to the proposed spatiotemporal neural network with attention, “*STANet*”, each result of blue, black, and green solid lines and bar graphs shows the cast-off using GRU instead of stateful LSTM, not using the attention mechanism, and not using RFB for encoding globally spatial information, respectively.

When the results of the ablation experiments were compared comprehensively using a combination of individual network modules, spatiotemporal feature representation with extended receptive field (RFB) provided the greatest performance improvement on the El Niño forecasts. It means that the prediction of El Niño is affected by a larger area including its Nino3.4 region, and a long time of sequence signals. Although temporal feature learning with GRU and spatial feature learning with attention mechanisms both improve performance, it is clear that their effects are maximized when the area and sequence of spatiotemporal feature learning are expanded simultaneously.

## Discussion

The *STANet*, spatiotemporal neural network with attention, was proposed in this study for predicting extreme events by learning spatiotemporal feature representations from geophysical data, and its performance was evaluated by applying it to El Niño forecasts. The architecture of the *STANet* has a wider receptive field with trainable attention for globally extended spatial feature encoding and more long-term memory for temporal sequential feature encoding.

When compared to the SOTA model, the proposed *STANet* for the multitasks of Nino3.4 index time series prediction on 12-month forecast lead time and the corresponding calendar month classification for El Niño forecasts in the test period of 1980–2017 improved the performance by 5.8% and 13%, respectively.

In addition, it showed a 10% improvement in performance for 9-month forecast lead forecasts compared to the SOTA model in the “spring predictability barrier” experiment. Furthermore, when the predicted time series of the Nino3.4 index for strong El Niño, an extreme event that occurred during the test period, was examined, it was confirmed that the peak value was predicted more accurately than the SOTA model.

Trained attention map allows interpreting the precursors for El Niño and La Niña occurrences for extreme ENSO events through the visualization of the trained attention map, which has the ability to figure out which of the spatial region to focus on. The attention map displays the representative El Niño precursor in the spring season, where the HC in the equatorial region and SST in the North Pacific are known as the Pacific Meridional Mode pattern. As a representative of the Central Pacific El Niño event in 2009, the attention map captures the precursor, North tropical Atlantic (NTA) SST signal. In general, La Niña has more diverse precursors than El Niño. La Niña could be linked to a wide range of developmental patterns. This may be related to a wide variety of La Niña developmental patterns. Overall, the result is also in good agreement with the climate dynamics for ENSO evolution.

In comparison to previous deep learning-based El Niño forecasting models that discovered patterns and trends buried within the data, it is believed that the proposed *STANet* understands the spatiotemporal behavior of global SST and HC as well as the nonlinear spatiotemporal relationship related to the mechanism of ENSO occurrence. There are also previous studies that showed good skills in the statistical model developed for medium- and long-range ENSO prediction^30,31^ with a correlation coefficient of 0.5 or more in 12-month and 24-month ahead prediction, respectively. While the proposed *STANet* was trained with historical simulation data of CMIP5, the above two statistical models were fitted with the National Centers for Environmental Prediction/National Center 40-year Reanalysis^32^ data and OISST v2 observation data, respectively. Therefore, it is difficult to directly compare the prediction performance of each ENSO forecasting, but it seems that it will be necessary to make an effort to improve the long-range prediction accuracy of deep learning approaches as well.

In Fig. 5, both neural networks underestimate large changes in Nino3.4 after 12 months. The uncertainties of the climate parameters and the neural networks are a possible explanation on the difficulty of the longer lead-time prediction. The uncertainties increase in the variance of the prediction^33^. Ensemble approaches using multiple models are promising in reducing the uncertainties for the Nino3.4 prediction^12^. Another reason for the limited performance is the lack of training data set enough for the neural networks. Due to the nature of the spatial-temporal climate data, conventional image augmentation methods, such as flipping, rotation, and translation, cannot be used for training the neural networks for the Nino3.4 index prediction. Generation of climate data using generative models, such as generative adversarial networks, is a possible option for the issue.

Time series extreme event forecasting in Earth science research is difficult task because it is rare or invisible in the training data gathered from historical observations and it is dependent on numerous external factors that can include complex weather-ocean interaction, not only in the top-down approach of the physics law-based numerical model, but also in the data-driven bottom-up approach, machine learning or deep learning method.

Deep learning, on the other hand, has the advantage of fast and cheap inference or prediction on new data after it has been trained. Speed and economy offer a distinct advantage over many physical models in Earth science, which must be inversely solved and which require significant time and computational resources for each application. Furthermore, recent advances in deep learning techniques such as adversarial generative networks, parameterization of phenomena and emulation, and physics-aware machine learning, are yielding very promising results in estimating atmospheric convection, SST and vegetation dynamic modeling^34^.

Therefore, as a future study, we intend to develop a network architecture allowing physical constraint based on physical laws and secure applicability to predict extreme weather, climate, and ocean phenomena. Above all, we are developing an explainable architecture that can quantitatively explain the rationale for the inference result to increase the trustworthiness of the deep learning method’s use.

## Methods

The *STANet* performs two tasks simultaneously with multi-input and multi-output time series prediction for El Niño forecasts based on given 3-month sequences of SST and HC anomalies: (1) SST anomaly prediction over the Nino3.4 region (Nino3.4 index) for a 23-month forecast lead time and (2) calendar month classification for the given input and predicted output. The reason why SST and HC are used as inputs is that it is well known that ocean HC resides a memory for the future ENSO evolution. In addition, the SST pattern is important because it modulates equatorial wind variability, which is a key to the ENSO evolution^7,35,36^.

For sequential SST anomaly prediction from $t+1$ to $t+23$, the *STANet* has a novel architecture based on a stateful LSTM with a spatiotemporal attention mechanism. During the learning process for SST anomaly prediction, the calendar month classification assists the *STANet* learning distinguishable features between seasonal inputs. For long-sequence Nino3.4 indices prediction, we also employed a correlation loss, smooth *L*1 loss, and cross-entropy loss in model training. In the sections that follow, we will first introduce the *STANet* architecture and will then go over the implementation details of its submodules for multitask learning.

### Model architecture

**Figure 9:**
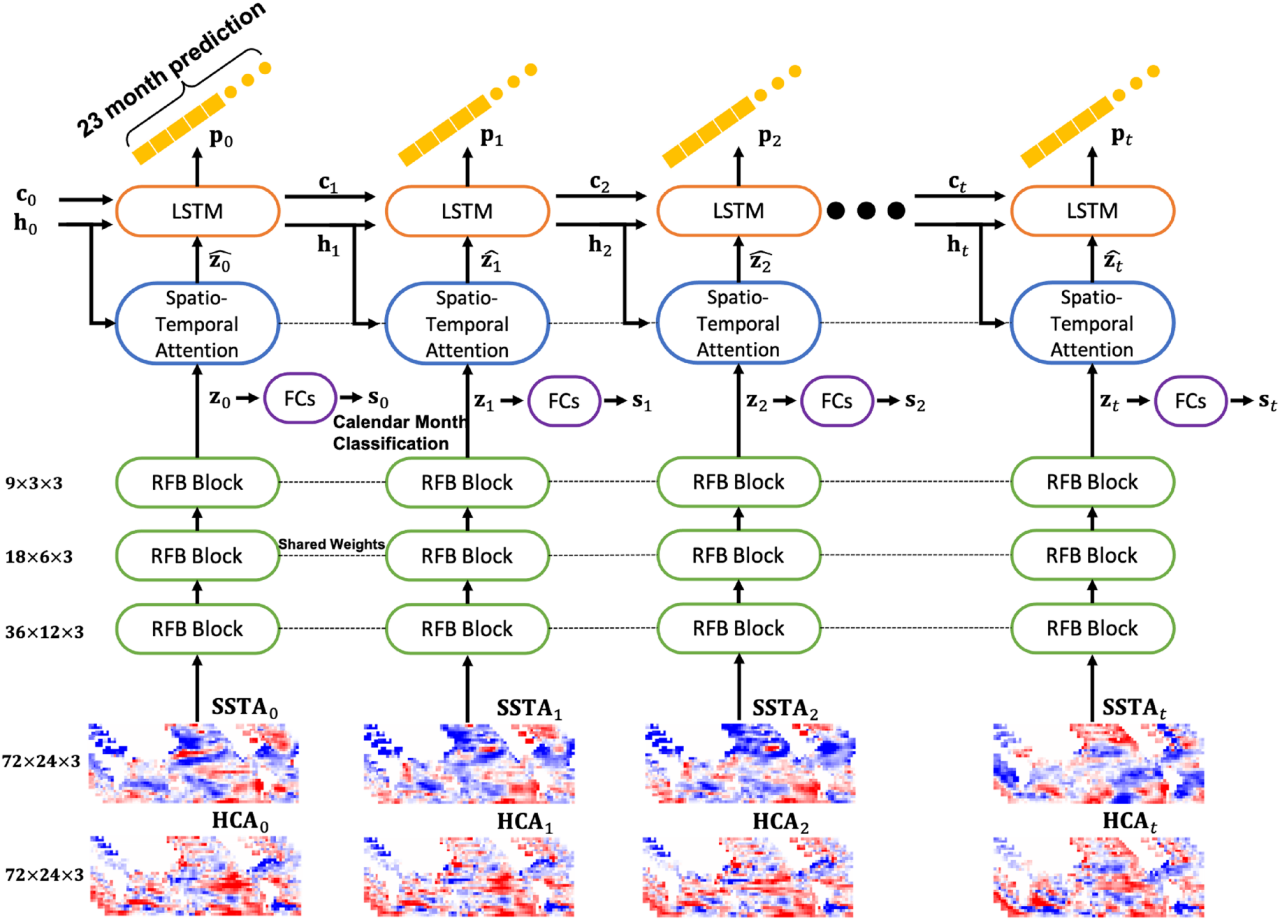
Architecture of the proposed *STANet*. “FCs” means two sequential fully-connected layers with ReLU and softmax functions respectively for the calendar month classification. “RFB Block” is a receptive field block with 3D convolution layers. “LSTM” is the stateful long short-term memory module.

The *STANet* consists of three modules for the multitask learning of SST anomaly prediction and calendar month classification. Figure 9 presents the entire architecture of the *STANet*. The first module of the *STANet* is a spatiotemporal encoding module that uses three-dimensional (3D) convolution layers with varying filter sizes, dilation factors, and residual connections to capture relevant features of given inputs in spatial and temporal domains. The second module is a stateful LSTM with a spatial attention mechanism to predict the 23-month SST anomaly values for long input sequences from the latent features of the encoding module. The final module is a classification module with two fully connected layers that are followed by hyperbolic tangent and softmax activation functions.

The input of the *STANet* consists of a 3-month consecutive input sequence of global SST and HC anomaly maps that are concatenated along feature dimensions. As a result, the input is a $T \times G_{lon} \times G_{lat} \times W \times F_{init}$ tensor where $G_{lon}$ denotes the size of the longitude grids and $G_{lat}$ denotes the size of the latitude grids. *W* is 3 as the observation window size of a given input. $F_{init}$ is the number of features, which is 2 for the SST and HC anomaly maps. *T* indicates the length of training or prediction period. In our approach, we used the stateful LSTM to give the network sequential inputs for the entire prediction period at once to understand long-term dependencies along time steps. For training, *T* was 32 consecutive months, which was determined through a grid search^37^. The *STANet* provides two outputs: consecutive 23 Nino3.4 indices for 23-month forecast lead time and a calendar month for given input sequences. Thus, the predicted Nino3.4 indices output is represented as a $T \times L$ matrix, where *L* is the total forecast lead time (23 months), and the classification output is a $T \times 12$ matrix. The calendar month for each input is determined as the month of the maximum probability. Figure 9 shows the prediction process of the *STANet* for the prediction period.

### Spatiotemporal encoding module with receptive field blocks

**Figure 10:**
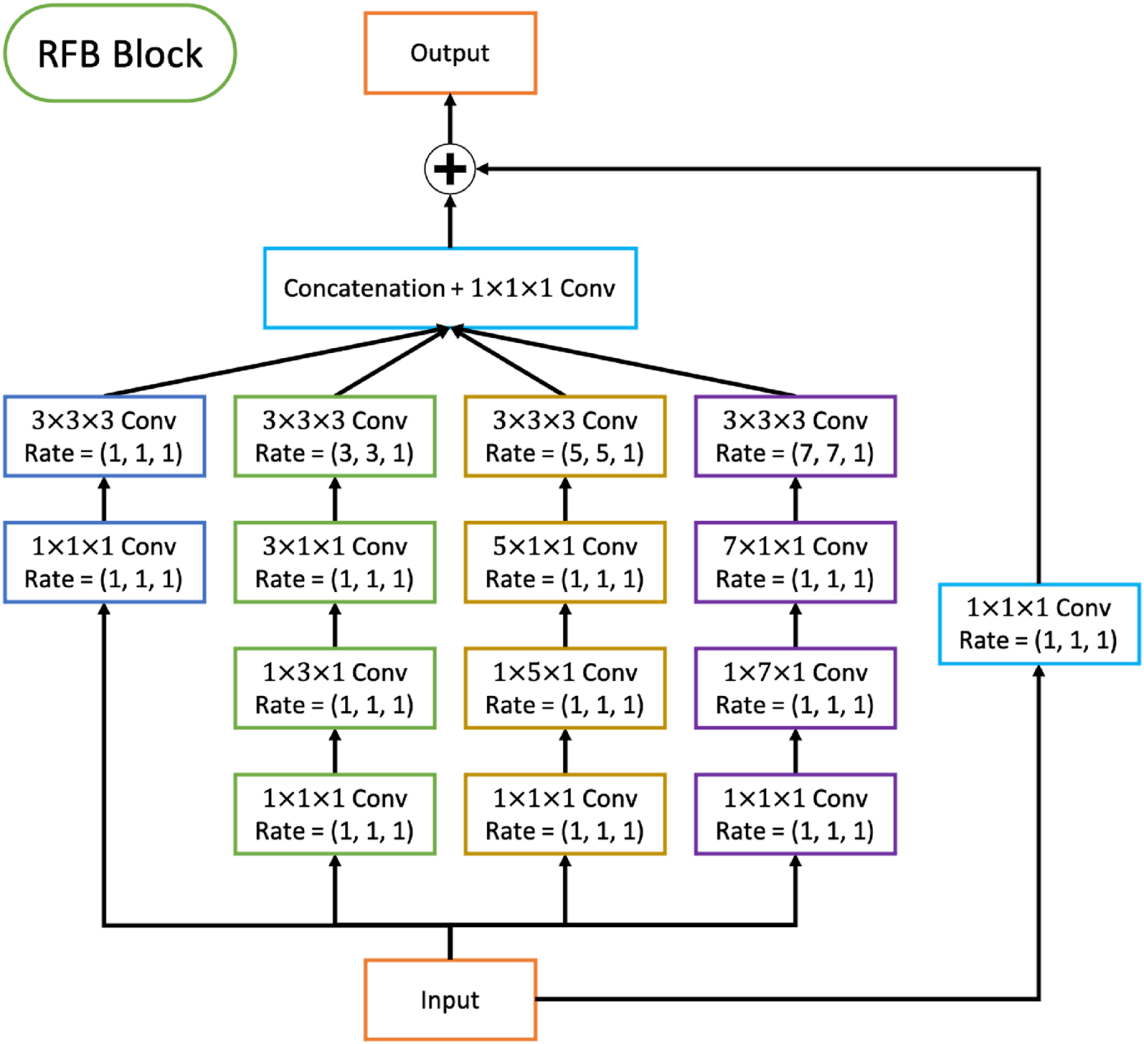
Structure of the receptive field block (RFB). “Conv” denotes convolution layer followed by ReLU activation function. “Rate” is the dilation rate of defining a spacing between the values in 3D kernels. The RFB includes a residual skip-connection to train deeper networks.

To extract the salient features of the given SST and HC anomaly maps for multitask learning, a 3D convolutional sub-network, named spatiotemporal encoding module, takes the first step of the *STANet*. In particular, rather than connecting several simple convolution layers as suggested in^12,15^, a receptive field block^38^ (RFB) is introduced into the module to learn spatial features globally by extending the receptive field of the network efficiently. Figure 10 shows the detailed structure of the spatiotemporal encoding module and RFB.

As inspired by the receptive field of the human visual system, an individual RFB includes multi-branch convolutional pooling with varying kernels corresponding to the receptive field of different sizes. Because the *STANet* receives inputs in 3D space (longitude, latitude, and time), we implement a 3D RFB with 3D convolutional layers. In the RFB, dilated convolutional layers with various dilatation rates from 1 to 7 are assigned to each branch. Because the observation window (*W*) is only 3, we consistently apply dilation rate 1 for the temporal dimension. The final representation of the latent features is then produced by concatenation following $1 \times 1 \times 1$ convolution layers in all branch (see Fig. 10). All convolution layers in the RFB are followed by a ReLU activation function. Each RFB block is followed by max-pooling with a kernel size of $2 \times 2 \times 1$ and strides (2, 2, 1) to downsample the feature maps. The *STANet* encodes the spatiotemporal features of SST and HC anomaly inputs, denoted as ${\mathbf {z}}_t$, by sequentially stacking three RFBs with max-pooling and applying a flatten operation for the spatial and temporal dimensions to be given to the stateful LSTM module and classification module. ${\mathbf {z}}_t$ is represented in $E \times F_{enc}$ dimension, and for the entire prediction period, the *STANet* provides *T* spatiotemporal feature vectors. Here, $F_{enc}$ indicates the number of spatiotemporal features.

**Figure 11:**
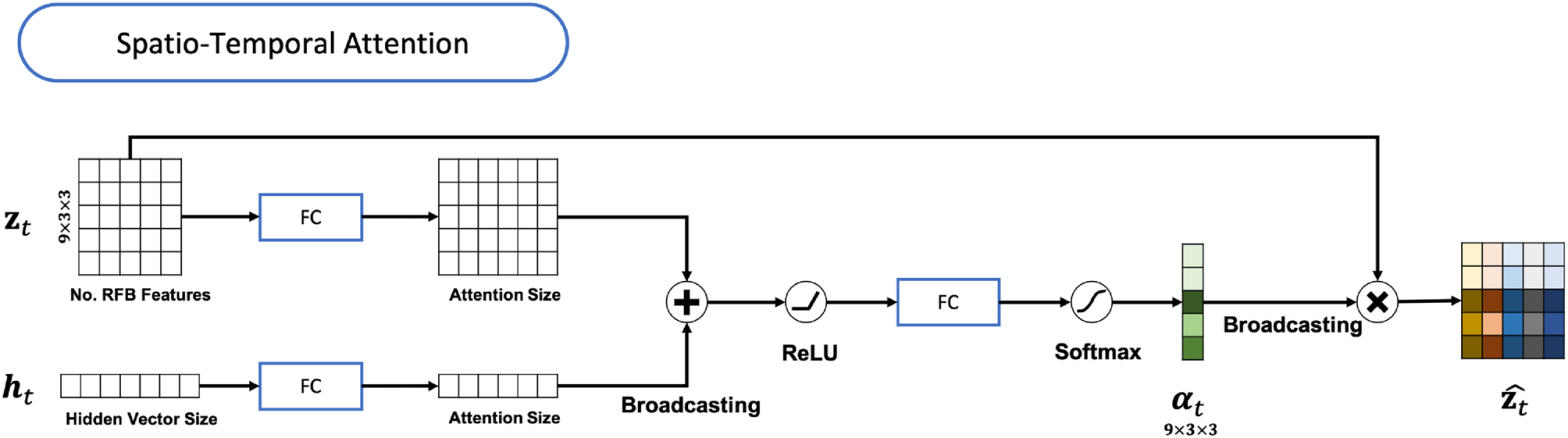
Structure of the trainable attention Module. “FC” and “Broadcasting” denote a fully-connected layer and implicit copying of vector, respectively.

### Stateful LSTM module with attention mechanism

We implemented a stateful LSTM sub-network (stateful LSTM module) inspired by a video captioning LSTM network with visual attention^39^ to predict the Nino3.4 indices of next *L* months for the prediction period (*T*) from the spatiotemporal features. At every time step during *T*, this module predicts *L*-dimensional vectors of Nino3.4 index predictions conditioned on a context vector and the previous hidden state. The operations of the LSTM cell are defined as follows:

1 $$\begin{aligned} \begin{aligned} {\mathbf {i}}_t&= \sigma (W_{zi}\hat{{\mathbf {z}}_t} + W_{hi}{\mathbf {h}}_{t-1} + W_{ci} \odot {\mathbf {c}}_{t-1} + b_i) \\ {\mathbf {f}}_t&= \sigma (W_{zf}\hat{{\mathbf {z}}_t} + W_{hf}{\mathbf {h}}_{t-1} + W_{cf} \odot {\mathbf {c}}_{t-1} + b_f) \\ {\mathbf {c}}_t&= {\mathbf {f}}_t \odot {\mathbf {c}}_{t-1} + {\mathbf {i}}_t \odot tanh (W_{zc}\hat{{\mathbf {z}}_t} + W_{hc}{\mathbf {h}}_{t-1} + b_c) \\ {\mathbf {o}}_t&= \sigma (W_{zo}\hat{{\mathbf {z}}_t} + W_{ho}{\mathbf {h}}_{t-1} + W_{co} \odot {\mathbf {c}}_{t} + b_o) \\ {\mathbf {h}}_t&= {\mathbf {o}}_t \odot tanh ({\mathbf {c}}_t) \end{aligned} \end{aligned}$$

where ${\mathbf {i}}_t,~{\mathbf {f}}_t,~{\mathbf {c}}_t,~{\mathbf {o}}_t,~{\mathbf {h}}_t$ are the input, forget, memory, output, and hidden state at time step *t*, respectively. In each equation, $W_*$ and $b_*$ are affine transformations with learned parameters and biases. The sigmoid function and Hadamard operation are denoted by the symbols $\sigma$ and $\odot$, respectively. The initial ${\mathbf {h}}_t$ and ${\mathbf {c}}_t$ ($t=0$) are computed from two fully-connected layers ($f_{h}$ and $f_{c}$) as follows:

2 $$\begin{aligned} \begin{aligned} h_0&= f_h(\frac{1}{E}\sum ^E_{i=1}z_{0,i})\\ c_0&= f_c(\frac{1}{E}\sum ^E_{i=1}z_{0,i}) \end{aligned} \end{aligned}$$

where ${\mathbf {z}}_{t, i}$ is a $F_{enc}$-dimension vector, $i=1, \cdots , E$, of ${\mathbf {z}}_t$ at different spatial locations and observation time ($1, \cdots , W$).

In Eq. (1), $\hat{{\mathbf {z}}_t}$ is the context vector which is the attentive context representation of ${\mathbf {z}}_t$ by an attention mechanism^39,40^. The attention mechanism, denoted as $\phi$, computes $\hat{{\mathbf {z}}_t}$ as follows:

3 $$\begin{aligned} \hat{{\mathbf {z}}_t} = \phi (\{{\mathbf {z}}_{t, i}\},\{\alpha _{t,i}\}) \end{aligned}$$

$\alpha _{t,i}$ is weight for each ${\mathbf {z}}_{t, i}$, determined by an simple attention model ($f_{att}$, see Fig. 11), which is a multi-layer perceptron conditioned on the previous hidden state (${\mathbf {h}}_{t-1}$).

4 $$\begin{aligned} \begin{aligned} s_{t, i}&= f_{att}({\mathbf {z}}_{t, i}, {\mathbf {h}}_{t-1}) \\ \alpha _{t,i}&= \frac{s_{t, i}}{\sum ^E_{j=1}s_{t, j}} \\ \phi (\{{\mathbf {z}}_{t, i}\},\{\alpha _{t,i}\})&=\beta _t \sum ^E_{i=1}\alpha _{t,i}{\mathbf {z}}_{t, i} \end{aligned} \end{aligned}$$

Here, $\beta _t$ is a gating scalar which is obtained by a fully-connected layer with sigmoid function from the previous hidden state ($h_{t-1}$). This attention mechanism encourages the *STANet* to focus on a relevant spatial location and observation time for Nino3.4 index prediction while understanding the contextual changes as well as the input sequences for the prediction period. Figure 11 shows the entire structure of the attention mechanism.

In the practical implementation of the *STANet*, *T* consecutive input sequences are given to a network as a minibatch. To learn the characteristic patterns of consecutive inputs, the *STANet* processes them sequentially without random indexing separately and saves the LSTM network states.

### Losses for *STANet*

The *STANet* is trained in end-to-end manner with three losses: (1) smooth *L*1 loss, (2) Pearson correlation loss, and (3) cross-entropy loss. The first two losses are employed for sequential SST anomaly prediction in the prediction period. The last loss is for calendar month classification. The smooth *L*1 loss assists the *STANet* minimizing the differences between the predicted Nino3.4 index and ground truth:

5 $$\begin{aligned} {\mathscr {L}}_{1} = \left\{ \begin{array}{ll} \frac{1}{TL}\sum ^T_{t=1}\sum ^L_{l=1} 0.5({\mathbf {y}}_{l,t}-{\mathbf {p}}_{l,t})^2/\beta ,&{} if \left| {\mathbf {y}}_{l,t}-{\mathbf {p}}_{l,t} \right| <\beta \\[6pt] \frac{1}{TL}\sum ^T_{t=1}\sum ^L_{l=1} \left| {\mathbf {y}}_{l,t}-{\mathbf {p}}_{l,t} \right| - 0.5*\beta ,&{} otherwise \end{array} \right. \end{aligned}$$

where $y_{l,t}$ is an observed SST anomaly at *l*-th month from the input month (*t*) and $p_{l,t}$ is the predicted SST anomaly of the corresponding month. The smooth *L*1 loss is less sensitive to outliers than the mean squared error loss, and it helps the network learn the dominant trends of SST anomaly along time. The Pearson correlation loss is used to increase the correlation between $Y_t$ and $P_t$ which are 23-dimension vector for observing and predicting SST anomalies, respectively. The correlation loss is defined as:

6 $$\begin{aligned} {\mathscr {L}}_{corr} = 1.0 - \frac{1}{T}\sum ^T_{t=1}\frac{ \sum ^L_{l=1}({\mathbf {y}}_{l,t}-\bar{{\mathbf {Y}}_t})({\mathbf {p}}_{l,t}-\bar{{\mathbf {P}}_t})}{\sqrt{\sum ^L_{l=1}({\mathbf {y}}_{l,t}-\bar{{\mathbf {Y}}_t})^2}\sqrt{\sum ^L_{l=1}({\mathbf {p}}_{l,t}-\bar{{\mathbf {P}}_t})^2}} \end{aligned}$$

The cross-entropy loss is for the calendar month classification for each input:

7 $$\begin{aligned} {\mathscr {L}}_{ent} = -\frac{1}{T}\sum ^T_{t=1}\sum ^{12}_i s_{i,t}log (\hat{s_{i,t}}) \end{aligned}$$

where $s_{i,t}$ is 1 for the corresponding calendar month of each input and 0 for others. $\hat{s_{i,t}}$ is the output of the softmax function. The *STANet* is trained using the weighted sum of all losses:

8 $$\begin{aligned} {\mathscr {L}}_{all} = \alpha {\mathscr {L}}_{1} + \beta {\mathscr {L}}_{corr} + \gamma {\mathscr {L}}_{ent} \end{aligned}$$

where $\alpha$ is 0.7, $\beta$ is 0.2, and $\gamma$ is 0.2 in this study.

## Experiments

### Data

Three kinds of datasets were used: historical simulation, reanalysis, and observation data as described in Table 1. The model was trained using historical simulation data generated by the 21 Coupled Model Intercomparison Project Phase 5 (CMIP5) model. Monthly mean SSTs from 1861 to 2004 with $5^{\circ } \times 5^{\circ }$ for the global area ($0^{\circ }$–360$^{\circ }$ E and 55$^{\circ }$ S–60 $^{\circ }$N) were used as input data for model training. El Niño occurs when the 3-month running mean of SST anomaly over the Nino3.4 region (170$^{\circ }$–120$^{\circ }$ W and 5$^{\circ }$ S–5$^{\circ }$ N), also known as the Ocean Nino Index exceeds 0.5 for at least 5 consecutive months. Thus, the Nino3.4 index is the area-averaged SST anomaly over the Nino3.4 region and is used as labeling data for supervised learning. The Nino3.4 index is a common index of ENSO. Besides SST anomaly, HC anomalies are also used as input data to train the network, which is the energy absorbed by the ocean.

**Table 1:** Description of datasets.

Item	Dataset	Period
Training	CMIP5 (historical simulation)	1861–2001
Validation	SODA (reanalysis)	1871–1970
Test	GODAS (reanalysis)	1980–2017
Comparison	OI SST v.2 (observation)	1982–2017

For model validation and testing, the monthly mean SST and HC produced from the reanalysis data of Simple Ocean Data Assimilation version 2.2.416 (SODA) for 1871–1970^41^ and the Global Ocean Data Assimilation System (GODAS) for 1980–2017^42^ are used.

Although the data periods are overlapped between SODA/GODAS and CMIP5, there is no dependency between the two datasets. In CMIP5 simulation, only long-term radiative forcing such as greenhouse gases and aerosols are prescribed, and it does not affect individual El Niño events, which have an interannual time scale. For example, the observed ENSO index and the simulated one show almost no correlation, suggesting they are completely independent. Furthermore, as the observation data for the Nino3.4 index, Optimum Interpolation SST version 2 (OISST v2) of the period 1982–2017 is used to compare the predictions^43^.

### Model implementation

Two input variables of SST and HC anomalies with a length of input sequence of 3 consecutive months were used to train a network to predict the Nino3.4 index of forecast lead time ranging from 1 to 23 months from 21 CMIP5 datasets. We used the SODA data as a tuning set to find optimal parameters using early stopping. Using the GODAS datasets from 1980 to 2017, the proposed network’s performance in Nino3.4 index prediction and calendar month classification was evaluated.

The parameters of the proposed network were randomly initialized using orthogonal initialization^44,45^. The network is trained using the Adam optimizer with learning rate ($\eta$) 0.001, $\beta _{1}$ 0.9, and $\beta _{2}$ 0.999. For the data augmentation, we added random Gaussian noise with $\gamma$ 0.2 to the SST and HC anomaly input maps. Model training and all experiments were conducted on a workstation with NVidia TITAN XP (12 GB memory), Intel i9 CPU, and 48 GB main memory. The batch size was 32 for the training. The proposed network and algorithms were implemented using Python 3.6.9 and PyTorch 1.8.1.

### Experimental design

To validate the effectiveness of the proposed methods for SST anomaly prediction over the Nino3.4 region, we performed ablation studies using the GODAS dataset as described in Table 2. First, we constructed three models without the *STANet*’s key components: RFB blocks for expanding the receptive field, an attention mechanism for focusing on relevant spatiotemporal features, and an LSTM module for learning the temporal patterns of input sequences for the prediction period. For the first model, we only replaced the RFB blocks with simple 3D (longitude, latitude, and time) convolution layers with the same activation functions as the RFB blocks. The second model was implemented only by removing the attention module from the *STANet*. We built the last model by changing the LSTM module to a gated recurrent unit (GRU)^29^. The GRU has updated and reset gates to solve the vanilla recurrent neural network’s vanishing gradient problem. Because the GRU outperformed the LSTM in learning long-term sequences on smaller and less frequent datasets, we chose it for the ablation study to validate the efficacy of the proposed method with the LSTM in learning long-term sequences.

For a comparison study with a state-of-the-art model, we built a *2D CNN* model^15^ using the authors’ TensorFlow codes, available at https://github.com/jeonghwan723/A_CNN. The *2D CNN* consists of three 2D convolution layers, followed by hyperbolic tangent (tanh) activation function and max pooling layer, two fully-connected layers with tanh function, and two separate fully-connected layers for Nino3.4 index prediction and calendar month classification. For the calendar month classification, the *2D CNN* uses softmax function. The *2D CNN* was trained to reduce mean squared error loss and cross-entropy loss for the Nino3.4 index prediction and the calendar month classification, respectively. In this study, the *2D CNN* was trained using the same data as the same conditions for comparative evaluation with the proposed *STANet*.

**Table 2:** The proposed *STANet* and three cases of ablation experiments for comparative analysis on spatiotemporal representation learning according to feature encoding architecture.

Network architecture	Spatial feature encoding	Temporal feature encoding	Attention mechanism
*STANet*	3D RFB	LSTM	Yes
GRU	3D RFB	GRU	Yes
No attention	3D RFB	LSTM	No
No RFB	3D Conv	LSTM	Yes
